# *In vitro* study of alginate–gelatin scaffolds incorporated with silica NPs as injectable, biodegradable hydrogels

**DOI:** 10.1039/d1ra02744a

**Published:** 2021-05-06

**Authors:** Mojgan Ghanbari, Masoud Salavati-Niasari, Fatemeh Mohandes, Banafsheh Dolatyar, Bahman Zeynali

**Affiliations:** Institute of Nano Science and Nano Technology, University of Kashan P. O. Box. 87317–51167 Kashan I. R. Iran salavati@kashanu.ac.ir +98 31 55913201 +98 31 5591 2383; Department of Cell and Developmental Biology, School of Biological Sciences, College of Science, University of Tehran Tehran Iran

## Abstract

Porous substrates composed of biodegradable polymers and nanoparticles have found extensive use as three-dimensional (3D) scaffolds to regenerate damaged tissues through the incorporation of cells or growth factors. Here, injectable thermally responsive hydrogels based on SiO_2_ nanoparticles (NPs), alginate, and gelatin biopolymers, with possible utilization for cartilage tissue engineering, are introduced. The nanocomposites contain different amounts of SiO_2_ NPs for reinforcement and 1-ethyl-3-(3-dimethylaminopropyl)carbodiimide (EDC)/*N*-hydroxysuccinimide (NHS) for chemical crosslinking of polymer chains in the 3D hydrogel network. The cross-sectional structure of the hydrogels containing 0.25, 1.5, and 3.0% SiO_2_ NPs was observed by FE-SEM, confirming porous morphology with interconnected pores. Based on the rheometer analyses, by increasing the amount of SiO_2_ NPs, the mechanical strength of the gels can be found. In addition, *in vitro* biodegradation studies show that the hydrogels without SiO_2_ are more unstable than the hydrogels containing SiO_2_ NPs. *In vitro* biocompatibility of the products tested by MTT assay indicates that cell viability and attachment depend on the presence of SiO_2_ NPs.

## Abstract 2

Thermoresponsive oxidized alginate/gelatin/SiO_2_ NPs injectable hydrogels have great potential in cartilage tissue engineering due to low gelation time (120 s) and body temperature gelation (37 °C).

## Graphical abstract

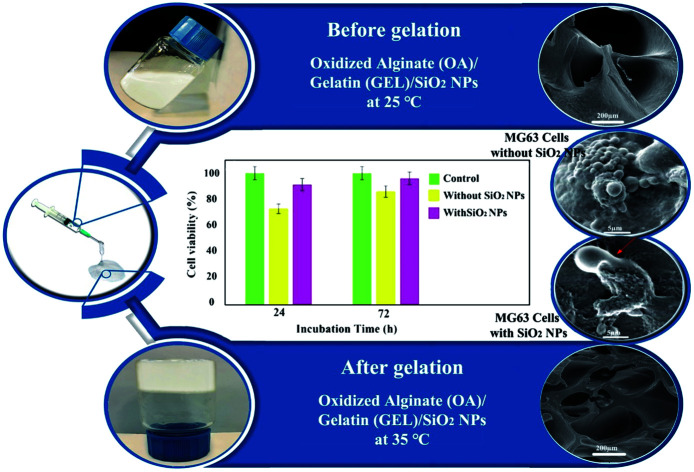

## 1. Introduction

Articular cartilage is generally injured due to degenerative joint disorders, for instance, osteoarthritis.^1,2^ Therefore, one of the most encouraging clinical obstacles for orthopedic doctors is the controlling of articular cartilage injuries.^3,4^ In such a manner, various surgical methods have been introduced to heal cartilage injuries. Since these endeavors have not been confirmed to be effective,^5–10^ accordingly, the current tissue engineering procedures are proposed as possible therapy alternatives to fix injured tissues or organs.^11,12^ Injectable hydrogels are three-dimensional (3D) networks with comparable attributes to the articular cartilage. They have been widely investigated as interim structures for cartilage reconstruction due to elasticity, unique biocompatibility, high porosity, absorbing and retaining a great quantity of water, hydrophilicity, and well- organized physical, chemical, and biological properties.^1,13–15^ The principal advantages of these hydrogels depend on their ability to adapt to the imperfection shape and to be effectively loaded down by cells or/and medicines alongside the development of growth factors and delivering cells to the imperfection region structure.^16–18^ Since the hydrogels which are interim scaffolds that mimic the extracellular matrix (ECM), hence the selection of the appropriate biomaterial to create these hydrogels is essential. Hydrogel scaffolds possess suitable mechanical characteristics and superior biocompatibility for improving tissue generation and cell adhesion.^19,20^ Many investigations have confirmed that the mechanical attributes of hydrogels perform an essential task in tissue reformation since they produce and save a place for cell generation.^21^ Hydrogel networks are usually formed with low mechanical strength, which prevents their usage as an aiding implant below load-bearing states. Therefore, the hydrogels usage has limitations to regenerate hard-tissue due to their weak mechanical characteristics.

Because most skeletal components of the body bear a part, the implanted or repaired section must be capable of carrying the least amount of pressure to keep the mechanical resistance of the implant section. Conventional hydrogels possess naturally poor mechanical stability below loading positions. Hydrogel's combination, which requires the inclusion of an inorganic reinforcement phase in the hydrogel, has been applied to defeat this shortcoming.^22^ Adding inorganic nanostructures in hydrogels causes mechanical improvement. It has been presumed that well-arranged nanostructures can strengthen the intermolecular aquaphobic relations *via* composing nanocomposite hydrogels and enhance the rheological performance of the hydrogels.^23^ Besides, nanostructures can reinforce the network structure of hydrogels, providing enhanced thermal and mechanical attributes. Until now, a small number of inorganic components have been studied, which comprise hydroxyapatite,^24,25^ layered double hydroxides,^26–28^ clay minerals,^29–31^ graphene oxide,^32,33^ metal oxide NPs,^34^ and carbon nanotubes.^35^ Silica nanoparticles (SiO_2_ NPs) and its surface modified nanocomposite, as multiple crosslinking agents, can create a group of robust nanocomposite hydrogels, which supply a facile and widely suitable approach for creating injectable and mechanical robust hydrogels.^36^

So far, natural polymers including alginate (AL),^37^ chitosan,^38^ hyaluronic acid,^39^ gelatin,^40^ and pectin^41^ have been investigated due to their resemblance to the ECM.^42–44^ Sodium alginate is a linear copolymer with blocks of (1–4)-α-l-guluronic acid (G) and (1–4)-β-d-mannuronic acid (M).^45^ This hydrogel possesses noteworthy characteristics, including excellent biodegradability and biocompatibility.^46^ Alginate has evolved into one of the most generally applied biological materials in injectable hydrogel formation for cartilage tissue engineering purposes due to its nontoxicity, non-immunogenicity, and suitable scaffold forming.^47,48^ Nevertheless, alginate is not strong enough to support the structural form of the regenerated tissue, and it is a shortcoming to utilize it as an injectable hydrogel.^49^ Hence, alginate is generally modified or applied in combination with other biological materials to enhance its mechanical characteristics. Oxidized alginate (OA) due to its multiple active functional groups (carboxylate and aldehyde groups) and a quicker degradation characterization than alginate has been attracted more attention for bio-applications.^50^ The polymeric chain is chemically modified by oxidation reactions on the –OH groups with potassium periodate (KIO_4_) to enhance the reaction features of natural alginate.^51^

Besides alginate, gelatin (GEL), a natural and biocompatible polymer, is wieldy used in medicinal treatments.^50^ GEL is a cationic polymer and easily creates hydrogels with OA or AL. Gelatin is a natural protein obtained from the degeneration of collagen with great biodegradability and biocompatibility in physiological conditions.^52,53^ Lately, the application of gelatin to fabricate injectable hydrogels has gained much attention. Nevertheless, GEL is dissolvable in water and unable to support mechanical pressure. Chemical crosslinking can overcome these shortcomings of gelatin.^50^ The combination of gelatin and alginate polymers as composite hydrogels exhibits excellent biocompatibility since OA and GEL are covalently bonded and can be ionically crosslinked. The biological fabrication of injectable hydrogels utilizing OA and GEL polymers still confronts challenges because a great concentration of OA and GEL is needed to accomplish the required porosity, mechanical strength, and viscosity.^54^ The main challenge of applying these hydrogels for tissue regeneration is their uncontrolled swelling, the inability of regeneration, degradation, and lack of ability to support 3D structures on their own. Bioceramics such as silicon dioxide nanoparticles (SiO_2_ NPs) are applied in combination with multiple polymers as a reinforcement to enhance the mechanical properties of the hydrogels.^55^ SiO_2_ NPs have free –OH groups on their surface which tend to form a hydrogen bond with COO– groups in biopolymers, including gelatin, agar, sodium alginate, and so on.^56^ Besides, it can be utilized for increasing growth factors or other bioactive molecules. The formation of a new hydrogen bond increases mechanical properties and enhances the hydrogel viscosity.^57^ Therefore, combining SiO_2_ NPs with OA and GEL hydrogels seems to be an encouraging answer to accomplish the required mechanical strength and viscosity for injectable scaffolds.

In this work, we selected substances that can imitate the cartilage properties: oxidized alginate (OA), gelatin (GEL), and ceramic silica nanoparticles (SiO_2_) as reinforcement. In the current study, hydrogel composites containing OA/GEL/SiO_2_ were fabricated by crosslinking the aldehyde groups of OA and the amino groups of GEL using 1-ethyl-3-(3-dimethylaminopropyl) carbodiimide (EDC) and *N*-hydroxysuccinimide (NHS) as chemical crosslinkers. We studied the impacts of oxidation of alginate on the mechanical and physical, morphological properties, and cytotoxicity of this hydrogel. We anticipate that this hydrogel creates a biodesign small-scale environment with high biodegradation and biocompatibility for repairing cartilage tissue.

## 2. Materials and methods

### 2.1. Materials

Sodium alginate (viscosity: 4–12 cP, 1% in H_2_O (25 °C) derived from brown algae, with molecular weight of 120 000–190 000 g mol^−1^), potassium periodate, gelatin (type B from bovine skin), *n*-propanol, ethyl alcohol, tetraethyl orthosilicate, sodium chloride, acetone, ethylene glycol, tetraethylpentamine, 1-ethyl-3-(3-dimethylaminopropyl) carbodiimide (EDC), *N*-hydroxysuccinimide (NHS), silver nitrate were purchased from Merck company and utilized without further purification.

### 2.2. Oxidation of alginate

2.01 g of sodium alginate and 11.2 mL of *n*-propanol were blended with DI-water in a 250 mL beaker to obtain 225 mL in total. The mix was kept at 30 °C in the dark under stirring (5 h) to dissolve alginate completely. 1.16 g of potassium periodate (KIO_4_) dispersed in 30 mL DI-water was combined with alginate solution. The mixture was kept in the dark for 24 h. The reaction was quenched by adding 1 mL of ethylene glycol (EG), and the mixture was agitated for another 30 min. 6.5 g of sodium chloride (NaCl) was dissolved in the above suspension to purify the polymer, which was next gently added to 400 mL agitated ethyl alcohol. The white precipitate was dissolved in DI-water with 3.3 g of NaCl and re-precipitated in 250 mL ethyl alcohol. The precipitate was dissolved in DI-water again and precipitated in 200 mL acetone. Eventually, the precipitate was rinsed in agitated ethyl alcohol for 15 min, refined, and dried at 25 °C.^58^ The lack of periodate was controlled by combining 500 μL fractional of the dialyzate to 500 μL of a 1% silver nitrate solution, and assuring the nonexistence of any precipitate.^59^

### 2.3. Synthesis of silica nanoparticles (SiO_2_ NPs)

In brief, 2.0 mL of tetraethyl orthosilicate (TEOS) was added to 20.0 mL of ethyl alcohol. Next, tetraethylenepentamine (TEPA) solution was added dropwise to the above solution (pH adjusted on 10) under sonication for 20 min. The white precipitate was centrifuged and washed with ethyl alcohol tree times. The powder was calcined at 400 °C for 2 h.

### 2.4. Preparation of OA/GEL/SiO_2_ hydrogels

5 mL of 6 wt% of OA solution was agitated with 5 mL of 15 wt% of GEL at 37 °C. The cross-linker, including a mixture of 0.1 g EDC and 0.05 g NHS, was added to the above solution. The first gelation was observed in 4–5 s and kept at 37 °C, resulting in the creation of a perfect gel after 2 min. The final powder could be obtained by freeze-drying (Alpha 2, 4, Martin Christ, Germany) of the hydrogels at −80 °C for 24 h. Different weight percentages of SiO_2_ (3.0%, 1.5%, and 0.25%) was added to the 5 mL of 6 wt% of OA solution and agitated for 5 min. Next, 5 mL of 15 wt% of GEL was added to the suspension and stirred for another 5 min. The final solutions were mixed for 2 min by adding EDC and NHS as cross-linker agents. The samples were freeze-dried at −80 °C for 24 h.

### 2.5. Materials characterizations

Fourier transform infrared spectroscopy (Shimadzu Varian 4300 spectrophotometer) was utilized to investigate the chemical composition of oxidized alginate and the fabricated hydrogels applying KBr pellets in the wavenumber between 4000–400 cm^−1^. A field emission scanning electron microscopy (TESCAN MIRA 3 FE-SEM) was used to study the morphological and structural of lyophilized hydrogels. The lyophilized hydrogels were cross-sectioned, covered by gold (Au), and detected by FESEM at an accelerating voltage of 15 kV. High-resolution transmission electron microscopy (EM 208, Philips HR-TEM with an accelerating voltage of 100 kV) was utilized to observe SiO_2_ NPs. A Physica MCR 300 Rheometer (Anton Paar Ltd., Austria) was utilized to measure the oscillatory rheological characteristics of the hydrogels.

### 2.6. Swelling ratio and biodegradation

The water absorption of hydrogels was evaluated by the gravimetric technique. About 0.3 g (*W*_0_) of the hydrogels were incubated in 10 mL PBS for 24 h to attain equilibrium swelling. The buoyant was eliminated, and the weight of swollen hydrogel was measured (*W*_s_):^19^1
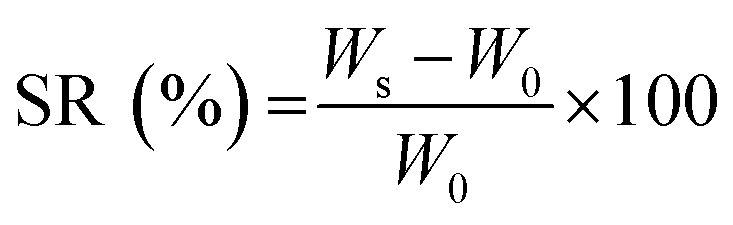


Mass degradation/erosion degrees were additionally evaluated likewise at various periods up to 21 days. All tests were accomplished three times.

### 2.7. Mechanical properties

A Physica MCR 300 Rheometer (Anton Paar Ltd., Austria) was used to measure the rheological attributes of the hydrogels utilizing a circular disk parallel plate with a diameter of 25 mm and a gap of 0.5 mm. An amplitude sweep was conducted at a consistent angular frequency of 1 Hz to define the limit of linear viscoelasticity. The strain amplitude was kept at 0.1% during the test. The contribution of the liquid-like form (viscous modulus (*G*′′)) and solid-like form (elastic modulus (*G*′)) were noted through temperature sweep from 20 to 50 °C at a speed of 1 °C min^−1^ to assess thermogelling attributes (angular frequency = 1 Hz). Each following rheological test was conducted below simulated physiological states (in PBS pH = 7.4 at 37 °C), considering the possible utilization of hydrogels. The oscillatory rheological determination as a function of time was conducted at a consistent frequency of 1 Hz to evaluate the time of gelation. The gel point or gelation time was specified as the time that the loss modulus and shear storage modulus were identical.^60^ The hydrogels were swollen for 1 h in 1 mL PBS and moved to the rheometer stage for performing crosslinked hydrogels. Next, frequency sweep analyses in the linear viscoelastic area were performed to determine the dynamic viscoelasticity at 37 °C on a broad range of frequencies (0.1–100 Hz).

### 2.8. *In vitro* biological assays

*In vitro* biocompatibility of the hydrogels was estimated utilizing 3-(4,5-dimethylthiazol-2-yl)-2,5-diphenyltetrazolium bromide (MTT assay), which depends on the mitochondrial MTT reduction to produce an insoluble dark blue formazan production. The samples were incubated in 1 mL of RPMI 1640 culture medium (Sigma-Aldrich) at 37 °C supplied by 10% (w/w) fetal bovine serum (FBS) for 24 and 72 h to achieve the extracts of the as-dried hydrogels. The growth medium (RPMI and FBS) was utilized as the control under similar conditions. The MG63 cells were cultivated in 96-well plates at a density of 1 × 10^4^ MG63 cells per sample. The growth medium was substituted by the hydrogels extract. The extract was removed after 24 h. 100 μL of the MTT solution (0.5 mg mL^−1^) was added to all wells and incubated for another four hours at 37 °C. Then, the solution was eliminated, and 100 μL isopropanol was consequently added to liquefy the MTT crystals. The absorbance of the solutions was measured with a microplate spectrophotometer (Biotek Powerwave XS2, USA) at 570 nm.

In order to study the architecture of the cell-attached to the hydrogels, cross-section SEM images of the samples have been recorded. The hydrogels were put in a Petri dish, and incubated in the existence of DMEM and MG63 cells at 37 °C for 24 h. After incubating, the hydrogels were rinsed multiple times by PBS and set by 2.5% glutaraldehyde solution for 4 h at 4 °C. Eventually, the samples were lyophilized and coated with Au for FESEM surveys.

## 3. Result and discussion

### 3.1. Oxidation of alginate

**Fig. 1:**
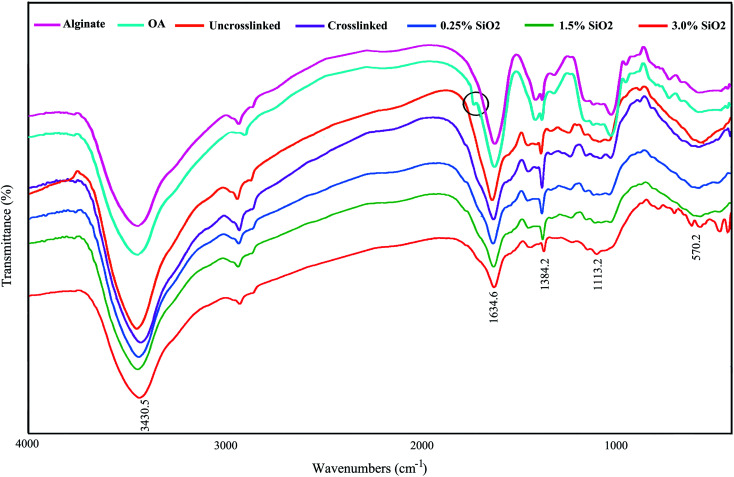
FTIR spectrum of alginate, oxidized alginate, and the hydrogels.

Fig. 1 displays the FTIR spectra of AL, OA, and the hydrogels comprising SiO_2_. The FTIR confirmed the presence of aldehyde groups (–CHO) on OA chains (Fig. 1). The characteristic bands at 1384 cm^−1^ and 1634 cm^−1^ are also being in OA, which are allocated to the symmetric and asymmetric carboxyl (COO) stretching modes on the AL structure, sequentially.^61^ Therefore, the oxidation reaction by KIO_4_ did not change the carboxyl groups in alginate. The new band at 1726 cm^−1^ in the OA exhibits the presence of aldehyde groups (–CHO). This peak is not identified in some cases owing to the hemiacetal configuration of hydroxyl groups with free aldehydes groups on nearby d-glucuronic acid subunits.^62,63^ The –OH stretching frequency is found at 3430 cm^−1^. The absorption band at 1634 cm^−1^ designates the C=N vibration of gelatin, confirming the creation of Schiff's base. As seen in FTIR spectra, a little shift is evident in the absorption band of OA in the OA–GEL cross-linked hydrogel. Moreover, the CHO group peak of OA at 1726 cm^−1^ has disappeared, and a new peak appeared at 1634 cm^−1^ is attributed to C=N bond.^64^ This band is due to the Schiff-base reaction in the amine group of GEL and the aldehyde group of OA,^65^ which confirmed that the cross-linking of GEL and OA transpired. The absorbton band at ∼1080 cm^−1^ is attributed to asymmetric stretching mode of (Si–O–Si), and stretching mode of Si–O–H is located at ∼985 cm^−1^.^66^

### 3.2. TEM images of SiO_2_

**Fig. 2:**
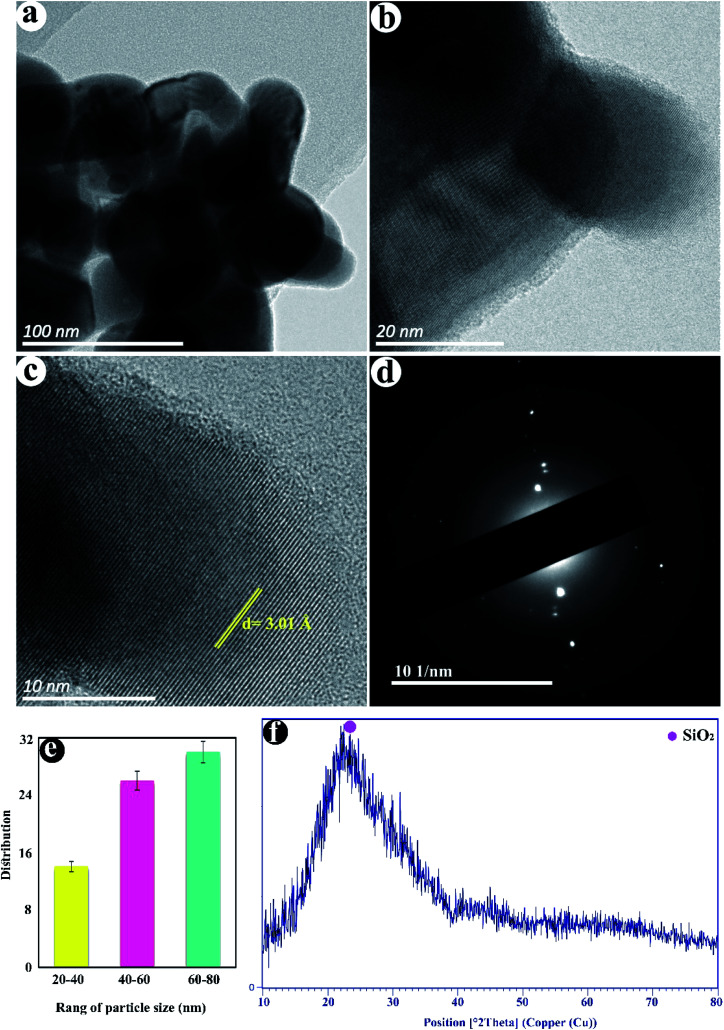
(a–c) TEM images, (d) SAED, (e) size distribution, and (f) XRD pattern of SiO_2_ NPs.

Fig. 2a–d displayed the TEM images of as-fabricated SiO_2_. The nanoparticles with a size distribution of 27–78 nm with an average diameter of ≈53 nm are observed in (Fig. 2e). The crystalline lattice plane with an inter-planar distance of 3.01 Å is observed corresponding to the (101) lattice plane of SiO_2_ (Fig. 2b and c). The SAED pattern in Fig. 2d shows the semi-crystalline structure of SiO_2_ NPs. The XRD pattern of SiO_2_ NPs shows the broad and strong peak in the range of 2*θ* = 15–35° can be attributed to amorphous silica (Fig. 2f).

### 3.3. Rheological studies

**Fig. 3:**
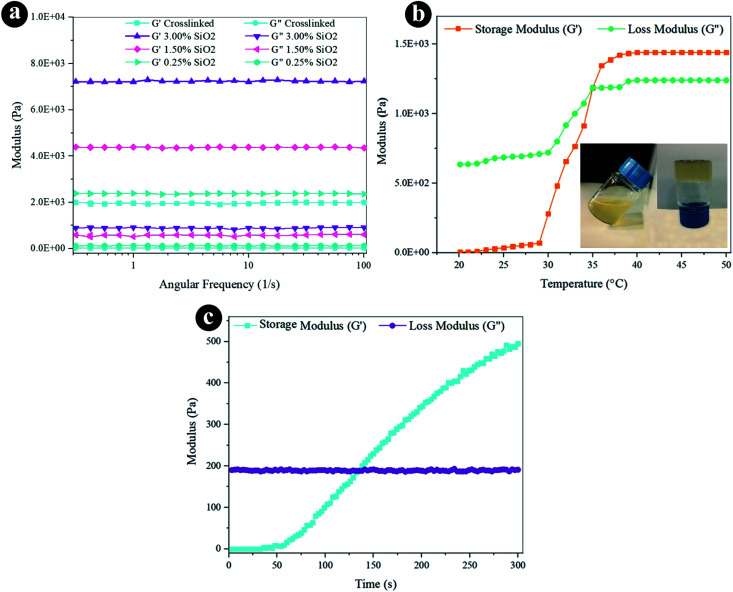
Rheological properties of the hydrogels by (a) frequency sweep, (b) temperature sweep, and (c) time sweep.

The rheological characteristics were conducted *via* oscillatory rheology to obtain knowledge on the stability of 3D crosslinked networks. The frequency sweep analyses of reinforced hydrogels were established at 37 °C (Fig. 3a), and the outcomes were displayed as loss modulus (*G*′′) and storage modulus (*G*′). Storage modulus was always higher than loss modulus for all the hydrogels, which indicates a durable crosslinked system. *G*′ developed quickly by increasing the weight percentage of SiO_2_, as presented in Fig. 3a and Table 1. The hydrogel containing 3.0% SiO_2_ indicates 3.6 fold greater degree of *G*′ correlated to the crosslinked hydrogel. Increasing the amount of SiO_2_ can direct to the greater crosslinking degree since it helps the mechanical improvement and the gel formation with the presence of a lot of reactive groups. The increased storage modulus (*G*′) for hydrogel containing higher amount of SiO_2_ NPs may also be due to the tight bonding of silica with the free COO^−^ and OH^−^ functional groups in the alginate and gelatin polymer network.

**Table 1:** Rheological properties of the hydrogels at 37 °C and frequency of 1 Hz

Sample	Storage modulus (Pa)	Loss modulus (Pa)	Average pore size (μm)
Crosslinked	1972 ± 32	24.6 ± 1.6	207.8
0.25% SiO_2_	2370 ± 20	124.7 ± 1.7	197.4
1.5% SiO_2_	4375 ± 15	564 ± 54	153.2
3.0% SiO_2_	7245 ± 45	870 ± 49	88.1

The oscillatory rheometry was applied to circumscribe the temperature of gelation of the conjugated crosslinked hydrogel with 0.1 g EDC and 0.05 g NHS by estimating *G*′ and *G*′′ *vs.* temperature at an angular frequency of 1 Hz. The temperature was raised of 20 to 50 °C through a speed of 2 °C min^−1^. The *G*′′ and *G*′ values were sketched *versus* temperature in Fig. 3b. The storage modulus depicts the flexible segment of the viscoelasticity, which is low at the liquid-like phase and grows substantially at the gelation temperature. The modulus values have gently raised as the temperature increases to 30 °C. The region where *G*′ is higher than *G*′′ exhibits that the elasticity is predominant, and in the area wherein *G*′ is lower than *G*′′, the viscosity is prevalent owing to hydrophobic interplay extension. The crossing spot of *G*′′ and *G*′ is the gelation temperature (35 °C) estimation and is frequently designated as the sol–gel transformation temperature.

Besides thermogelling performance, gelation time is an important feature of the injectable hydrogel structure. Injectable hydrogels require to maintain liquid within surgical processes and injections, but when injected, they must rapidly turn to gel.^67^ We can control the formation of the hydrogel with the improvement of the viscoelastic behavior of the substance at the gel point, wherever the transmutation of the fluid-like to the solid phase appears. Hence, the gel point is described as the *G*′ and *G*′′ crossover.^60^ The progression of the crosslinked hydrogel of *G*′′ and *G*′ moduli was established as a function of time within gel creation at 37 °C (Fig. 3c). *G*′ is lower than *G*′′ before gelation, which presents predominant viscous characteristics and a fluid-like behavior at the beginning of the gelation. The *G*′ rate increases faster than the *G*′′ at longer times. It means the fluid-like phase has become a more solid gel with predominant elasticity. As the chemical crosslinking agent is injected, stable covalent systems slowly substitute the physical chain complexes of polymer chains that enhance in *G*′ over time. As the reaction proceeds, the *G*′ and *G*′′ converge at the gel point.^68^ Certainly, EDC/NHS can perform as *in situ* covalent crosslinking agents in gelation. The gelation time is determined at 120 s for the composite hydrogel from the time sweep analysis outcomes. The fleeting gelation time is adequate for the injection of the composite hydrogels.

### 3.4. Microstructure of hydrogels

**Fig. 4:**
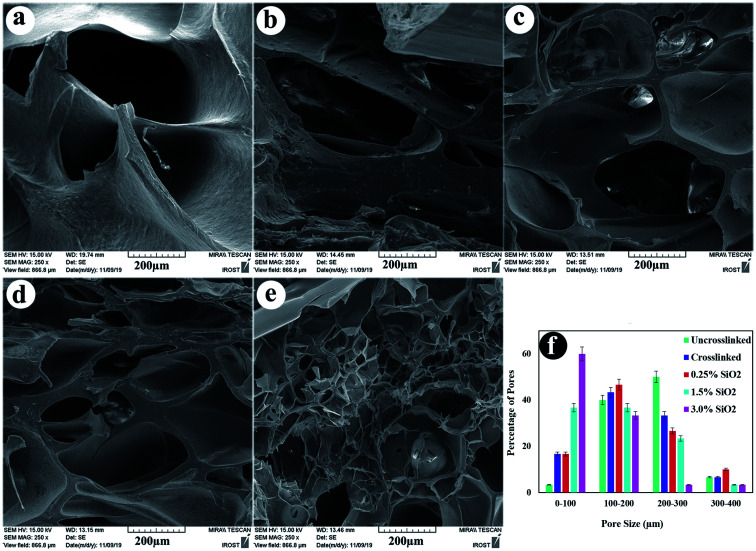
Cross-section morphology of freeze-dried hydrogels (a) uncrosslinked, (b) crosslinked, (c) containing 0.25% SiO_2_, (d) 1.5% SiO_2_, (e) 3.0% SiO_2_, and (f) pore size distribution of the samples.

The microstructure morphology of hydrogels is also essential because it regulates the recovery of tissues, helping the delivery of biological portions and mass transfer in the hydrogel system.^69^ The cross-sectional structures of the uncrosslinked, crosslinked hydrogels, and their composites containing 0.25, 1.5, and 3.0% SiO_2_ were observed by FE-SEM (Fig. 4). The FESEM images reveal porous and uniform scaffold structure with variable form and the average pore sizes between 88–207 μm, which suited for cartilage regeneration.^70^ The compositional unity shows good coordination among the ingredients in the nanocomposite hydrogels. The morphology of the hydrogels in Fig. 4 unveil that the pore size decreases lightly by combining crosslinkers in the hydrogel. This conclusion indicates the variation in the crosslinking density, which is significantly higher in the crosslinked hydrogels. The addition of SiO_2_ as reinforcement has directed to a reduction in the microstructures pore size. By increasing the concentration of SiO_2_ NPs in the alginate–gelatin hydrogel mixture, the free OH groups on the SiO_2_ surface promote further bonding sites for the formation of hydrogen bond within SiO_2_ and gelatin as well as SiO_2_ and sodium alginate. In this case, although all the hydrogel groups are crosslinked by EDC/NHS, the hydrogels with a higher amount of SiO_2_ show smaller pore sizes. This designates the further degree of crosslinking obtained *via* the hydrogen bonding between SiO_2_ and OA/GEL polymer network.^54^

### 3.5. Swelling and degradation

**Fig. 5:**
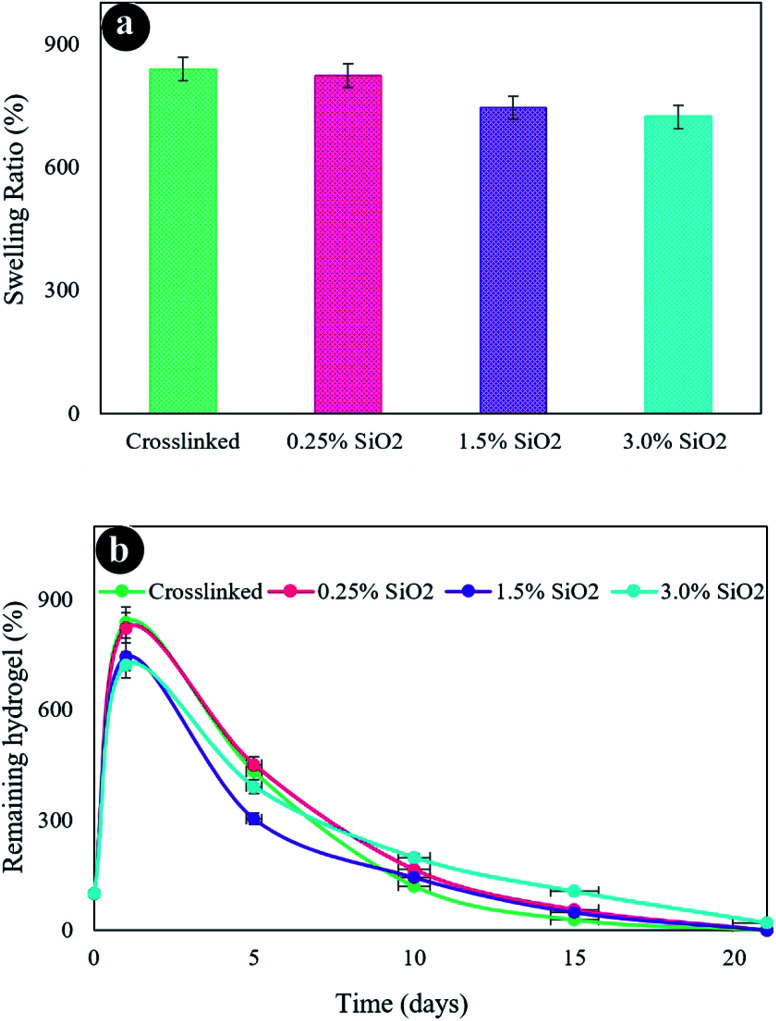
(a) Swelling ratio of the hydrogels, (b) *in vitro* biodegradation after various incubation times in PBS at 37 °C.

The swelling properties of freeze-dried hydrogels were assessed in PBS solution at 37 °C after 24 h (Fig. 5a). Hydrogels possess an excellent water uptake capability owing to their hydrophilicity and high porosity. The hydrogel without SiO_2_ unveils the highest swelling rate of 838.2%. The swelling has somewhat diminished at higher SiO_2_ content owing to the smaller pore sizes in other hydrogels, which decreases the water uptake. The outcomes are harmonious amidst FESEM images. The hydrogel containing a high amount of SiO_2_ (3.0%) exposed more compressed pore size and crosslinked networks associated with the hydrogel containing less amount of SiO_2_ (0.125%), which was directed to less water absorption and consequently less swelling degrees. Accordingly, it can be deduced that the swelling characterization of hydrogels principally is dependent on the density of crosslinker.^1^ An increment in the crosslinking density was obtained due to the formation of more covalent bonding in the hydrogel networks. Therefore, the movement of the hydrogel network chains was decreased by the addition of SiO_2_ into the hydrogel matrix. Thereby the swelling capability is decreased.^71,72^

Fig. 5b presents the in *vitro* degradation of hydrogels. As displayed in this figure, the crosslinked hydrogel (without SiO_2_) was more quickly decomposed than the other fabricated hydrogels. The constant rise in the degradation of the hydrogel composition was recognized by enhancing submersion time after 21 days. The hydrogel without SiO_2_ gave a weight loss considerably higher than the other hydrogels after 21 days of incubation. The hydrogels with a varied amount of SiO_2_ present a similar degradation process that has the same degradation index. Therefore, a greater decomposition degree of hydrogels was perceived with lower SiO_2_ contents, which might be because of the reduced network crosslinking density. It ought to be noted that the rate of scaffold degradation is reduced in water after cross-linking and can be utilized as an extracellular matrix (ECM) to maintain the cell culture media.

### 3.6. Cell viability and attachment

**Fig. 6:**
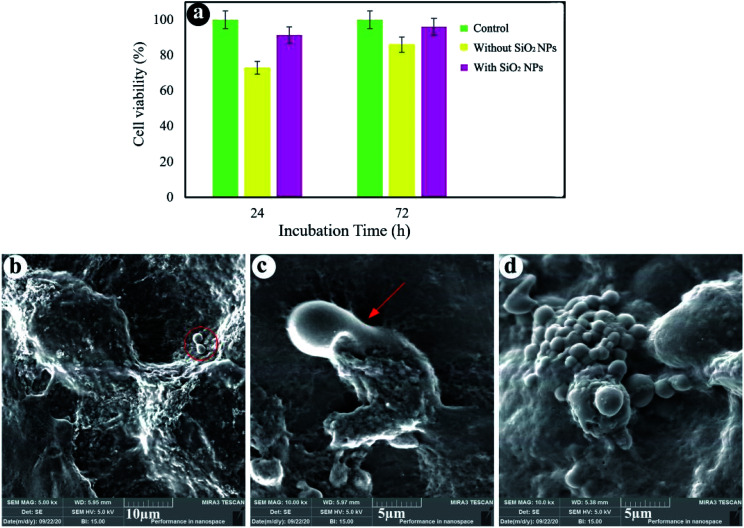
Cell viability (a), FE-SEM images of cell-cultured hydrogels with 3.0% SiO_2_ (b and c) and without SiO_2_ (d).

Fig. 6a shows that cell viability of the sample without SiO_2_ NPs after 24 and 72 h of cultivation is close to 73% and 86%, respectively. Moreover, the hydrogels containing 3.0% SiO_2_ NPs show 91% and 96% cell viability after 24 and 72 h of incubation. By comparing the results, it was found that SiO_2_ NPs can promote the cell growth and viability. As shown in Fig. 6a, cell viability of the hydrogels is less than that of the control test, since the behavior of cells depends strongly on the cell density seeded on their surface and porous structure of the hydrogels.^73^ When the cells are spread on the walls of the inner pores of the hydrogels, reducing the cell proliferation.^74^ However, the cells seeded at high densities (more than 1.0 × 104 cells) on the porous substrates cause the fluctuation of cell proliferation as time goes on. As cells can fill the pores quickly, reducing cell proliferation owing to cell contact inhibition of growth. A few days later, the time required to colonize a new pore, cell proliferation could be repeatedly perceived.^75^Fig. 6b and c show the SEM images of cells seeded on the hydrogels after 24 h cell culture. We can see that the cells were stuck to the surface of the hydrogels containing 3.0% SiO_2_ NPs, showing efficient interplays among the surrounding hydrogel and cells. The existence of filopodia spread from the cells to the porous substrate (Fig. 6c) designates that the cells were well-attached to the hydrogels. Although the presence of the cells can be observed on the hydrogel surface synthesized without NPs, the spherical morphology of the cells without any filopodia indicates no effective interaction between the cells and hydrogel. As a results, cell attachment can be also improved by using the SiO_2_ NPs in the chemical composition of the hydrogels.

## 4. Conclusion

In summary, thermal responsive hydrogels containing SiO_2_ NPs, alginate and gelatin biopolymers have been fabricated *via* simple precipitation and freeze-drying method. The effect of SiO_2_ concentration on the physical, chemical and biological properties of the composites was investigated. So by increasing the amount of SiO_2_ NPs from 0.25% to 3.0%, the mechanical strength, chemical stability in the simulated body fluid as well as cell growth increased. The fleeting gelation time of the nanocomposites is adequate for the injectable hydrogels, introducing a potential candidate for cartilage tissue engineering. Therefore, these composite hydrogels have great potential and scope for their application in nanomedicine and tissue engineering. This research of confirming the injectable hydrogels containing SiO_2_ NPs opens the possibility of investigating the performance of SiO_2_. This procedure appears to be promising to make an impact in the health care industry.

## Conflicts of interest

There are no conflicts to declare.

## Supplementary Material
